# The mediatory effect of inflammatory markers on the association between a body shape index and body roundness index with cardiometabolic risk factor in overweight and obese women: a cross-sectional study

**DOI:** 10.3389/fnut.2023.1178829

**Published:** 2023-06-09

**Authors:** Atieh Mirzababaei, Faezeh Abaj, Darya Khosravinia, Moloud Ghorbani, Neda Valisoltani, Cain C. T. Clark, Mina Radmehr, Khadijeh Mirzaei

**Affiliations:** ^1^Department of Community Nutrition, School of Nutritional Sciences and Dietetics, Tehran University of Medical Sciences, Tehran, Iran; ^2^Department of Nutrition, Science and Research Branch, Islamic Azad University, Tehran, Iran; ^3^Department of Community Nutrition, Faculty of Nutrition and Food Sciences, Tabriz University of Medical Sciences, Tabriz, Iran; ^4^Department of Clinical Nutrition, School of Nutritional Sciences and Dietetics, Tehran University of Medical Sciences, Tehran, Iran; ^5^Centre for Intelligent Healthcare, Coventry University, Coventry, United Kingdom

**Keywords:** a body shape index, body roundness index, inflammation markers, obesity, cardiometabolic

## Abstract

**Background:**

Obesity affects body composition and anthropometric measurements. A Body Shape Index (ABSI) and Body Roundness Index (BRI) are reportedly associated with an increased risk of cardiovascular disease. However, the relationship between ABSI, BRI, cardiometabolic factors, and inflammatory elements is not well-elucidated. Therefore, this study sought to examine the mediatory effect of inflammatory markers on the association between ABSI and BRI with cardiometabolic risk factors in overweight and obese women.

**Methods:**

This cross-sectional study was performed on 394 obese and overweight women. The typical food intake of individuals was assessed using a 147-item semi-quantitative Food Frequency Questionnaire (FFQ). Body composition was measured by bioelectrical impedance analysis (BIA). Biochemical parameters, such as inflammatory markers and anthropometric components, were also assessed. For each participant, all measurements were carried out on the same day.

**Result:**

There was a significant positive association between ABSI and AC and CRI.I in subjects with higher ABSI scores before and after adjustment (*P* < 0.05). In addition, there was a significant positive association between BRI and FBS, TC, TG, AIP, AC, CRI.I, CRI.II, and TyG in participants with higher BRI scores before and after adjustment (*P* < 0.05). We found that hs-CRP, PAI-1, MCP-1, TGF-β, and Galectin-3 were mediators of these relationships (*P* < 0.05).

**Conclusion:**

Inflammation can play an important role in the relationship between body shape indices and cardiometabolic risk factors among overweight and obese women.

## 1. Introduction

Worldwide, the incidence of overweight and obesity represents a significant public health issue. Globally, in 2016, 39% of men and 40% of women, aged 18 years and older, representing almost 2 billion adults, were overweight, and 11% of men and 15% of women, over half a billion, were obese (1). The prevalence of obesity tends to increase with age and is typically greater in the women population (2). Obesity, broadly speaking, occurs when energy intake goes beyond energy utilization for a long period of time, resulting in fat accumulation (3). Genetic, social, environmental, cultural, and biological elements play an important role in obesity development. One of the most plausible causes of global increases in weight is the biological response to low-cost and energy-rich food, combined with an extensive decrease in physical activity levels (4, 5). Being overweight and obese have been related to an increased risk of cardiovascular disease (CVD), metabolic syndrome (MetS), dyslipidemia, hypertension, stroke, type 2 diabetes, many types of cancer, such as colon and postmenopausal breast cancers, osteoarthritis, and other musculoskeletal disorders (6). CVD is one of the most evidence-based clinical implications of obesity (7); indeed, the CVD mortality rate is directly and indirectly influenced by obesity. Indirect impacts are due to concomitant CVD risk factors, such as insulin resistance, glucose intolerance, and high blood pressure, while direct effects are associated with inflammation (8–10).

Obesity and overweight directly affect body composition and, thus, anthropometric measurements. The body mass index (BMI), waist circumference (WC), and waist-to-height ratio (WHR) have been regularly used as anthropometric predictors of CVD for many years, and several studies have indicated distinct cardiovascular risk profiles in people with alike BMI. However, A Body Shape Index (ABSI) and Body Roundness Index (BRI) are reported to represent more accurate indicators (11–13). High ABSI and BRI are related to excess abdominal adipose tissue accumulation. Furthermore, BRI and ABSI are posited to be more sensitive to the presence of MetS, insulin resistance, and inflammatory factors as cardiovascular risk factors (14–16).

By considering the relationship between ABSI and BRI and cardiometabolic factors, inflammatory elements may play an effective role. Imbalanced lipid profiles and inflammatory factors, such as Galectin-3, monocyte chemotactic protein-1 (MCP-1), plasminogen activator inhibitor-1 (PAI-1), and transforming growth factor β (TGF-β,) which are related to body composition and obesity, play a significant role in increasing the risk of cardiovascular diseases (17). Galectin-3, a β-galactoside-binding protein belonging to the lectin family, is a novel predictor of heart failure (HF) risk and mortality. Other key processes, in which Galectin-3 plays a role, include inflammation, tissue fibrosis, and angiogenesis. Normally, Galectin-3 has a low expression in the heart, and its synthesis and secretion increase in HF. Galectin-3 primarily protects the heart with anti-apoptotic and anti-necrosis functions, but its long-term expression results in fibrosis and unfavorable regeneration of the damaged tissue. Galectin-3-binding sites are mainly in the myocardial matrix, fibroblasts, and macrophages (18, 19). Additionally, MCP-1 plays a major role in CVD development, and this protein, through its chemotactic activity, causes diapedesis of monocytes to the subendothelial space, where they are converted into foam cells and begin fatty streaks and finally give rise to atherosclerotic plaque formation. In addition, inflammatory macrophages probably are involved in plaque rupture and ischemia caused by it, as well as restenosis after angioplasty. There is strong evidence for a major role of MCP-1 in myocarditis, ischemia/reperfusion injury in the heart, and transplant rejection. Moreover, MCP-1 has various effects on the types of cells involved in cardiac fibrotic remodeling. Despite the multiple functions of MCP-1 in cardio-pathobiology, the molecular mechanisms underlying these functions are poorly understood (20). The likelihood of ischemic cardiovascular events is associated with PAI-1 levels, and the increase in PAI-1 levels raises the risk of atherothrombotic events and may also lead to the progression of vascular disease (21). In addition, TGF-β is reported to have a significant effect on the cardiovascular system. TGF-β is a cytokine that exerts a wide range of different and often contradictory functions. Its effects on the cardiovascular system are also ambiguous because, on the one hand, there is strong evidence for the “protective cytokine hypothesis” that expresses TGF-β as an anti-atherogenic factor, and on the other hand, it has been proven to be involved in some pro-inflammatory effects. Moreover, in addition to the beneficial and restorative positive role of TGF-β, there are disadvantages as follows: TGF-β plays an important role in postangioplasty restenosis and postinfarction myocardial remodeling (leading to heart failure), and it also plays a role in many blood circulation disorders that are related to fibrosis and vascular regeneration (22). Despite the above evidence, the relationship between ABSI, BRI, cardiometabolic factors, and inflammatory elements is not well-elucidated. Therefore, this study sought to examine the mediatory effect of inflammatory markers on the association between ABSI and BRI with cardiometabolic risk factors in overweight and obese women.

## 2. Methods

### 2.1. Study design and population

This cross-sectional study was carried out on 394 non-postmenopausal, healthy, overweight, and obese (BMI = 25–40 kg/m^2^) women, aged 18–48 years, who were referred to healthcare centers of Tehran University of Medical Sciences (TUMS). The multi-stage cluster sampling method was used for sampling. Participants who had any acute or chronic disease background, such as high blood pressure, diabetes mellitus, cancer, polycystic ovary syndrome (PCOS), and hepatic and kidney disease, were excluded. Other exclusion criteria included alcohol consumption, pregnancy and lactation, adherence to special/non-normal dietary intake, and substantial body weight changes in the last year. Furthermore, women using medications that affect body weight and/or glucose and/or lipid-lowering drugs were not eligible to participate in this study. We also excluded subjects whose energy intake was <800 or more than 4,200 (kcal/day) (as under-reporters and over-reporters) (23, 24). Written consent forms were obtained from each participant before the study commencement. The protocol of this study was approved by the Ethics Committee of the TUMS (IR.TUMS.VCR.REC.1397.804).

### 2.2. General and anthropometric assessment

General information, including age, occupational and educational status, and marital status, were collected from all participants. The patient's height was determined by a digital stadiometer (Seca Germany) while barefoot and reported to be the nearest 0.1 cm. Systolic blood pressure (SBP) and diastolic blood pressure (DBP) were measured by a standard mercury sphygmomanometer in the right arm after 15 min of rest in a sitting position. The first and fifth Korotkoff sounds were taken as SBP and DBP, respectively. The average of the two measurements is considered as the participant's blood pressure. Anthropometric measurements, such as weight, WC, and hip circumferences (HC), were evaluated by skilled technicians who followed standard guidelines. The midpoint between the lowest rib and iliac crest was used for measuring WC. WHR was computed by dividing WC by HC, and BMI was obtained by Equation 1 as follows:


(1) $$\begin{array}{l} BMI=\frac{Wt}{{Ht}^{2}} \end{array}$$

Where, Wt is the weight (kg) and Ht is the height (m).

Finally, BMI = 25–29.9 kg/m^2^ was considered overweight, and obesity was described as BMI = 30–40 kg/m^2^. A bioelectrical impedance analyzer (BIA) device (In Body 770, Korea) was used to assess body composition variables (25). BRI was calculated by equation 2, with the help of an online calculator (26):


(2) $$\begin{array}{l} BRI=364.2-365.5\;\times \;{\left(1-\;\frac{{\left[\frac{WC}{2\pi }\right]}^{2}}{\left[0.5\;\times \;{Ht}^{2}\right]}\right)}^{\frac{1}{2}} \end{array}$$

where WC is the waist circumference (cm) and Ht is the height (cm).

In addition, ABSI was computed by Equation 3 (27) as follows:


(3) $$\begin{array}{l} ABSI=\frac{WC}{\left[{BMI}^{\frac{2}{3}}\;\times \;{Ht}^{\frac{1}{2}}\right]} \end{array}$$

Where, WC is the waist circumference (cm), BMI is the body mass index (kg/m^2^), and Ht is the height (cm).

### 2.3. Biochemical assessment

Venous blood samples were collected from all participants following 12 h fasting. After centrifuging, liquating, and storing samples at −80°C at the TUMS nutrition laboratory, the following parameters were measured: fasting blood sugar (FBS) by using the Glucose Oxidase phenol 4-Aminoantypyrine Peroxidase (GOD/PAP) method, serum insulin values by utilizing radio-immune assay, lipid profile inclusive high-density lipoprotein cholesterol ( HDL), low-density lipoprotein cholesterol (LDL), total cholesterol (TC), and triglyceride (TG) by applying enzymatic procedure (Pars Azmun Co, Tehran, Iran). Hypersensitive C-reactive protein (hs-CRP), TGF-β, PAI-1, MCP-1, and Galectin-3 levels were measured by the enzyme-linked immunosorbent assay (ELISA) method. The atherogenic index of plasma (AIP), a useful parameter in clinical practice, was determined as the logarithm of the TG/HDL ratio. Additionally, we compute the lipid ratio based on the definition by Olamoyegun et al. (28), calculated as follows:


(4) $$\begin{array}{l} CRI-\text{I}=\frac{TC}{HDL-C} \end{array}$$

(5) $$\begin{array}{l} CRI-\prod =\frac{LDL-C}{HDL-C} \end{array}$$

(6) $$\begin{array}{l} AC=\frac{\left(TC-HDL-C\right)}{HDL-C} \end{array}$$

Moreover, the triglyceride glucose (TGy) index was calculated as follows (29):


(7) $$\begin{array}{l} TyG\;index=ln\left[\frac{\begin{array}{c} fasting\;triglycerides\;\left(mg/dL\right)\times \\ \;fasting\;plasma\;glucose\;\left(mg/dL\right) \end{array}}{2}\right] \end{array}$$

### 2.4. Physical activity assessment

The validated international physical activity questionnaire (IPAQ) was applied to collect data about participants' physical activities. Finally, metabolic equivalent (MET) scores and MET-minutes per week (MET-min/wk) were calculated as a summation of all activity categories and reported as mild (<600), moderate (600–3,500), and severe (>3,500) (MET-h/wk.) (30).

### 2.5. Dietary assessment

A 147-item Food Frequency Questionnaire (FFQ) was applied to assess usual dietary intake. The validity and reliability of FFQ were confirmed in Esmaillzadeh et al. study (31). The questionnaires were administered by an expert nutritionist, and all subjects were asked to report the frequency of different food items consumed during the previous year. Daily (e.g., bread), weekly (e.g., rice, meat), or monthly (e.g., fish) categories were considered for each item's report. By using household measurements, the frequency of each food consumption was changed into grams per day. In the next step, the Iranian Food Composition Table and N4 program were used to assess dietary nutrients and total energy intake.

### 2.6. Statistical analysis

The Kolmogorov–Smirnov test was used to examine the data's normal distribution. Quantitative and qualitative differences between the ABSI and BRI groups were described using independent *t*-test and chi-square test, respectively. In this study, participants were dichotomized based on their median ABSI and BRI scores (0.791 and 5.642, respectively). To describe the characteristics of the study population among the ABSI and BRI groups, we used analysis of covariance (ANCOVA), considering age and total energy intake as cofounders. We also used linear regression to investigate the association between ABSI and BRI with metabolic markers and atherogenic indices. BRI group <5.642 and ABSI group <0.791 were considered the reference groups. In this model, we also examined the mediating effects of inflammatory markers (hs-CRP, PAI-1, MCP-1, TGF-β, and Galectin-3). We created several models to examine the mediating role of these inflammatory indicators. First, we examined the crude and adjusted model, including age and energy intake. Then, in the final model, we separately included each of the inflammatory markers as a confounding variable along with age and energy intake. Finally, we considered the mediating effect of the variables. Data analysis was conducted using SPSS software (version 25, SPSS Inc., Chicago, IL, USA). *P* < 0.05 was considered statistically significant.

## 3. Results

### 3.1. Study population characteristics

This cross-sectional study was performed on 394 overweight and obese Iranian women. The mean age, weight, BMI, ABSI, and BRI of participants were 36.67 ± 9.10, 80.28 ± 11.05 kg, 30.98 ± 3.90 kg/m^2^, 0.79 ± 0.02, and 5.85 ± 1.35, respectively. Among the study population, 286 (72.4%) were married, 138 (35.1%) were employed, and 189 (47.8%) had a bachelor's degree or higher. It should be noted that 127 (49.2%), 119 (46.1%), and 12 (4.7%) participants had low, moderate, and high physical activity, respectively.

### 3.2. Characteristics of the study population among the ABSI and BRI groups

The mean and standard deviation (SD) of quantitative and qualitative variables among the ABSI and BRI groups are shown in Table 1.

**Table 1:** Characteristics of the study population among the ABSI and BRI groups.

**Variables**	**ABSI**				**BRI**			
	**Lower**<**0.791**	**Higher** ≥**0.791**	***P*** **-value**	***P*** **-value** ^*^	**Lower**<**5.642**	**Higher** ≥**5.642**	***P*** **-value**	***P*** **-value** ^*^
	**(*****n*** = **197)**	**(*****n*** = **197)**			**(*****n*** = **197)**	**(*****n*** = **197)**		
**Demographic**								
Age (years)	37.32 ± 8.88	35.75 ± 9.33	0.09	0.08	35.32 ± 8.72	37.83 ± 9.33	**0.006**	**0.006**
**Anthropometric parameters**								
Weight (kg)	77.96 ± 10.86	82.45 ± 10.47	**<0.001**	**<0.001**	73.77 ± 7.16	86.85 ± 10.07	**<0.001**	**<0.001**
Height (cm)	158.92 ± 5.45	163.36 ± 5.3	**<0.001**	**<0.001**	161.9 ± 10.56	160.34 ± 5.96	**0.008**	0.07
BMI (kg/m^2^)	30.88 ± 4.15	31 ± 3.57	0.76	0.16	28.09 ± 1.89	33.87 ± 3.12	**<0.001**	**<0.001**
WC (cm)	95.14 ± 8.57	102.74 ± 8.33	**<0.001**	**<0.001**	92.15 ± 5.52	106.15 ± 6.97	**<0.001**	**<0.001**
WHR	1.36 ± 6.54	0.96 ± 0.03	0.39	0.32	0.9 ± 0.03	1.43 ± 6.5	0.25	0.14
**Body composition**								
BFM (kg)	32.69 ± 8.09	35.34 ± 7.15	**0.01**	**<0.001**	28.63 ± 4.31	39.95 ± 6.8	**<0.001**	**<0.001**
BFP (%)	41.61 ± 5.76	45.31 ± 4.86	0.19	0.31	38.87 ± 4.16	45.27 ± 4.5	**<0.001**	**<0.001**
VFA (cm^2^)	162.16 ± 125.32	175.7 ± 32.14	0.14	0.26	147.41 ± 122.07	192.11 ± 27.71	**<0.001**	**0.003**
VFL	14.87 ± 3.43	16.76 ± 2.72	**<0.001**	**<0.001**	13.48 ± 2.54	18.26 ± 1.82	**<0.001**	**<0.001**
FMI	13.09 ± 3.44	13.27 ± 2.7	0.56	0.16	11.02 ± 1.81	15.56 ± 2.63	**0.04**	**<0.001**
**Blood pressure**								
SBP (mmHg)	110.87 ± 14.01	111.72 ± 12.65	0.59	**0.001**	109.59 ± 12.15	113.69 ± 14.79	**0.01**	0.07
DBP (mmHg)	77.2 ± 9.86	77.61 ± 10.89	0.74	0.09	76.38 ± 9.19	78.8 ± 11.46	**0.05**	0.13
**Job**								
Unemployed	112 (50.5%)	110 (49.5%)	0.10	0.10	98 (43.4%)	128 (56.6%)	**0.002**	**0.003**
Employed	78 (50%)	78 (50%)			94 (59.9%)	63 (40.1%)		
**Education**								
Illiterate and under diploma	31 (60.8%)	20 (39.2%)	0.18	0.17	18 (34%)	35 (66.0%)	**0.006**	**0.005**
Diploma and higher diploma	75 (51.4%)	71 (48.6%)			69 (46.6%)	79 (53.4%)		
Bachelor and higher	86 (46.5%)	99 (53.5%)			107(57.5%)	79 (42.5%)		
**Marital status**								
Single	54 (47.4%)	60 (52.6%)	0.50	0.45	58 (50.4%)	57 (49.6 %)	0.11	0.20
Married	141 (51.3%)	134 (48.7%)			139(49.9%)	140 (50.2%)		
**Metabolic markers**								
FBS (mmol/dl)	86.7 ± 9.67	87.9 ± 9.78	0.33	0.11	85.6 ± 7.61	89.46 ± 11.45	**0.002**	**0.002**
TC (mg/dl)	182.62 ± 33.9	185.49 ± 36.95	0.52	0.41	178.64 ± 31.05	191.95 ± 39.7	**0.003**	**0.003**
TG (mg/dl)	115.86 ± 56.78	118.73 ± 57.72	0.69	0.60	108.51 ± 52.14	131.34 ± 64.4	**0.002**	**0.002**
HDL (mg/dl)	47.54 ± 10.25	46.10 ± 11.25	0.30	0.43	47.07 ± 9.72	46.33 ± 11.98	0.57	0.59
LDL (mg/dl)	96.3 ± 23	94.03 ± 24.92	0.46	0.44	93.1 ± 22.1	98 ± 25.95	0.11	0.11
**Atherogenic indices**								
AIP	0.35 ± 0.23	0.37 ± 0.24	0.41	0.41	0.32 ± 0.22	0.42 ± 0.26	**0.004**	**0.004**
AC	2.96 ± 0.96	3.27 ± 1.77	0.09	0.09	2.91 ± 0.94	3.42 ± 1.87	**0.006**	**0.006**
CRI-I	2.08 ± 0.54	2.1 ± 0.6	0.09	0.09	3.91 ± 0.94	4.42 ± 1.87	**0.03**	**0.006**
CRI-II	3.96 ± 0.96	4.27 ± 1.77	0.71	0.87	2.03 ± 0.53	2.19 ± 0.61	**0.004**	**0.03**
TyG	1.33 ± 0.62	1.36 ± 0.67	0.73	0.77	1.27 ± 0.61	1.47 ± 0.71	**0.02**	**0.02**
**Physical activity**								
Low	55 (45.5%)	66 (54.5%)	1.922	1.875	64 (52.5%)	58 (47.5%)	0.762	0.761
Moderate	64 (54.2%)	54 (45.8%)			68 (57.6%)	50 (42.4%)		
High	5 (45.5%)	6 (54.5%)			6 (50.0%)	6 (50.0%)		

ABSI, A Body Shape Index; BRI, Body Roundness Index; SBP, systolic blood pressure; DBP, diastolic blood pressure; BMI, body mass index; WC, waist circumference; WHR, waist–hip ratio; BFM, body fat mass; BFP, body fat percentage; VFA, visceral fat area; VFL, visceral fat level; FMI, fat mass index; FBS, fasting blood sugar; TC, total cholesterol; TG, triglyceride; HDL, high-density lipoprotein; LDL, low-density lipoprotein; AIP, atherogenic index of plasma; AC, atherogenic coefficient; CRI, Cardiac Risk Index; TyG, triglyceride glucose.

*P*-values resulted from the independent *t*-test for continuous variables and the chi-square test for categorical variables.

Quantitative variables were presented as mean ± SD and qualitative variables as frequency (percentage).

*P*-value^*^ was adjusted for age and total energy intake. The bolded values are related to significant *p*-value = < 0.05.

Based on our findings, the means of some variables in the crude model, including weight, height, WC, VFL (*P* < 0.001), and BFM (*P* = 0.001), were significantly higher in subjects with higher ABSI scores than subjects with lower ABSI scores. The means of SBP (*P* = 0.01), height (*P* = 0.008), weight, BMI, WC, BFM, BFP, VFA, VFL (*P* < 0.001), FMI (*P* = 0.04), FBS (*P* = 0.002), TC (*P* = 0.003), TG (*P* = 0.002), AIP (*P* = 0.004), AC (*P* = 0.006), CRI.I (*P* = 0.03), CRI.II (*P* = 0.04), and TyG (*P* = 0.02) were significantly higher in subjects with higher BRI scores compared to those with lower BRI scores.

After we adjusted age and total energy intake as confounding variables, weight, height, WC, BFM, and VFL remained significantly higher in subjects with higher ABSI scores than in subjects with lower ABSI scores. The mean of weight, BMI, WC, BFM, BFP, VFL, VFA (*P* = 0.003), FMI (*P* = 0.04), FBS (*P* = 0.002), TC (*P* = 0.003), TG (*P* = 0.002), AIP (*P* = 0.004), AC (*P* = 0.006), CRI.I (*P* = 0.006), CRI.II (*P* = 0.032), and TyG (*P* = 0.02) remained significantly higher in participants with higher BRI scores compared to those with lower BRI scores. However, the mean height and systolic blood pressure (SBP), after adjustment for confounding factors, were not significantly different between subjects with higher BRI scores compared to subjects with lower BRI scores.

### 3.3. Dietary intakes among ABSI and BRI groups

The mean and SD of energy and dietary intakes of the participants among the ABSI and BRI groups are shown in Table 2. Although energy intake was higher in subjects with higher ABSI and BRI scores compared to those with lower scores, this difference was not significant. After adjusting for total energy intake as a confounding variable, we observed that selenium (*P* = 0.01) and B12 (*P* = 0.01) intakes were significantly higher in participants with higher ABSI scores, but vitamin C (*P* = 0.03) and sucrose (*P* = 0.02) intakes were significantly higher in participants with lower ABSI score. Among the BRI group, calcium (*P* = 0.03) and iron (*P* = 0.02) intakes were significantly higher in women with higher BRI scores. The mean intake of other macronutrients and micronutrients was not significantly different between the study groups (*P* > 0.05).

**Table 2:** Dietary intakes among the ABSI and BRI groups.

	**ABSI**			**BRI**		
**Variables**	**Lower**<**0.791**	**Higher** ≥**0.791**	***P*** **-value**	**Lower**<**5.642**	**Higher** ≥**5.642**	***P*** **-value**
	**(*****n*** = **197)**	**(*****n*** = **197)**		**(*****n*** = **197)**	**(*****n*** = **197)**	
**Macronutrients**						
Energy (kcal/day)	2621.03 ± 807.35	2729.57 ± 802.71	0.91	2559.95 ± 764.41	2695.21 ± 837.85	0.10
Protein (g/day)	90.45 ± 32.52	92.01 ± 29.94	0.47	87.66 ± 27.65	94.82 ± 33.41	0.11
Carbohydrate (g/day)	371.69 ± 121.64	371.07 ± 125.88	0.69	364.78 ± 122.01	379.18 ± 126.01	0.26
Fat (g/day)	94.51 ± 35.34	94.93 ± 3.94	0.96	91.59 ± 32.65	97.78 ± 36.15	0.50
**Subtypes of fatty acid**						
Cholesterol (mg/day)	259.28 ± 115.69	269.31 ± 11.43	0.34	258.89 ± 111.94	269.15 ± 114.61	0.99
SFA (g/day)	27.61 ± 10.55	29.01 ± 12.43	0.90	27.31 ± 1.12	29.24 ± 11.79	0.54
MUFA (g/day)	31.71 ± 2.92	31.87 ± 12.12	0.93	30.64 ± 11.6	32.91 ± 3.33	0.42
PUFA (g/day)	20.38 ± 9.86	19.51 ± 8.45	0.20	19.17 ± 8.29	20.7 ± 10	0.44
Trans fatty acid (g/day)	0.0001 ± 0.001	0.001 ± 0.002	0.06	0.0001 ± 0.002	0.0001 ± 0.002	0.55
**Micronutrients**						
**Minerals**						
Calcium (mg/day)	1242.75 ± 35.03	1299.04 ± 542.79	0.19	1196.61 ± 490.7	1339.87 ± 562.77	**0.03**
Iron (mg/day)	25.74 ± 19.29	27.37 ± 22.78	0.43	23.51 ± 15.37	29.44 ± 25.02	**0.02**
Magnesium (mg/day)	468.36 ± 169.86	482.57 ± 172.51	0.22	453.95 ± 150.07	497.31 ± 87.6	**0.05**
Zinc (mg/day)	13.19 ± 5.09	13.6 ± 4.57	0.17	12.81 ± 4.61	13.99 ± 5.02	0.06
Selenium (mg/day)	122.06 ± 46.14	130.55 ± 2.06	**0.01**	120.74 ± 44.16	132.27 ± 53.66	0.11
Phosphor (mg/day)	1644.52 ± 562.1	1702.82 ± 557.84	0.09	1609.94 ± 530.7	1737.37 ± 581.8	0.12
**Vitamins**						
C (mg/day)	197.81 ± 32.11	176.62 ± 93.97	**0.03**	187.31 ± 128.84	188.89 ± 104.16	0.45
D (μg/day)	1.82 ± 1.45	16.44 ± 8.85	0.08	1.93 ± 1.5	1.98 ± 1.61	0.81
E (mg/day)	17.45 ± 9.1	16.44 ± 8.85	0.20	16.42 ± 8.69	17.53 ± 9.39	0.60
B1(mg/day)	2.1 ± 0.7	2.16 ± 0.77	0.18	2.06 ± 0.88	2.21 ± 0.77	0.26
B2(mg/day)	2.23 ± 0.87	2.32 ± 0.85	0.16	2.2 ± 0.88	2.34 ± 0.83	0.66
B3(mg/day)	26.149.78	26.53 ± 10.3	0.62	25.230.81	27.47 ± 11.09	0.15
B6 (mg/day)	2.19 ± 0.76	2.19 ± 0.73	0.9	2.1 ± 0.68	2.28 ± 0.8	0.08
B_12_ (mg/day)	4.04 ± 2.2	4.58 ± 2.58	**0.01**	4.29 ± 2.59	4.35 ± 2.19	0.54
**Total Fiber** (g/day)	48.35 ± 19.03	46.63 ± 23.83	0.26	46.42 ± 20.85	48.41 ± 22.08	0.81

ABSI, A Body Shape Index; BRI, Body Roundness Index; SFA, saturated fatty acid;MUFA, monounsaturated fatty acid; PUFA, polyunsaturated fatty acid.

Variables were presented as mean ± SD.

Nutrients were adjusted for total energy intake. The bolded values are related to significant *p*-value = <0.05.

### 3.4. The association of ABSI with metabolic markers and atherogenic indices

The association between ABSI with metabolic markers and atherogenic indices is demonstrated in the crude and adjusted models in Table 3. In the crude model, there was a significant association between ABSI with AC (β = 0.13, 95% CI = 0.02–0.07, *P* = 0.03) and CRI-I (β = 0.13, 95% CI = 0.02–0.07, *P* = 0.03) in subjects with higher ABSI scores, as compared to those with lower ABSI scores. In addition, after adjusting for age and total energy intake, as confounding variables, in a general linear model, this association remained significant (β = 0.13, 95% CI = 0.02–0.07, *P* = 0.03). No significant association was observed between ABSI scores with other metabolic markers and atherogenic indices before and after adjustment (*P* > 0.05).

**Table 3:** Association of ABSI with metabolic markers and atherogenic indices.

**Metabolic markers**	**Models**	**ABSI higher** ≥**0.791**		
		β	**95% CI**	**P-value**
FBS (mmol/dl)	Crude	0.07	−1.03 to 3.85	0.25
	Adjusted	0.1	−0.33 to 4.58	0.09
TC (mg/dl)	Crude	0.04	−5.53 to 12.24	0.45
	Adjusted	0.05	−4.86 to 13.35	0.36
TG (mg/dl)	Crude	0.02	−11.57 to 17.31	0.69
	Adjusted	0.03	−11.18 to 19.09	0.6
HDL (mg/dl)	Crude	−0.18	−4.6 to 0.85	0.17
	Adjusted	−0.07	−4.47 to 1.18	0.25
LDL (mg/dl)	Crude	−0.05	−8.86 to 3.29	0.36
	Adjusted	−0.05	−8.53 to 3.43	0.40
**Atherogenic indices**				
AIP	Crude	0.05	−0.03 to 0.08	0.41
	Adjusted	0.05	−0.03 to 0.09	0.41
AC	Crude	0.13	0.02 to 0.7	**0.03**
	Adjusted	0.13	0.02 to 0.8	**0.03**
CRI-I	Crude	0.13	0.02 to 0.7	**0.03**
	Adjusted	0.13	0.02 to 0.8	**0.03**
CRI-II	Crude	0.04	−0.09 to 0.19	0.50
	Adjusted	0.02	−0.11 to 0.18	0.62
TyG	Crude	0.02	−0.13 to 0.19	0.73
	Adjusted	0.01	−0.14 to 0.19	0.77

ABSI, A Body Shape Index; FBS, Fasting Blood Sugar; TC, total cholesterol; TG, triglyceride; HDL, high-density lipoprotein; LDL, low-density lipoprotein; AIP, atherogenic index of plasma; AC, atherogenic coefficient; CRI, Cardiac Risk Index; TyG, triglyceride glucose.

Metabolic markers and atherogenic indices are adjusted for age and total energy intake in the linear regression model.

The ABSI group <0.791 was considered the reference group. The bolded values are related to significant *p*-value = <0.05.

### 3.5. The evaluation of the mediating effect of inflammatory markers in the relationship between ABSI and atherogenic indices

We investigated the effect of inflammatory markers including hs-CRP, PAI-1, MCP-1, TGF-β, and Galectin-3 as mediators for the association between ABSI and atherogenic indices (Table 4). Each of these inflammatory markers was included in the final model as a confounding variable along with age and total energy intake. We observed that some of these inflammatory markers attenuated the relationship between ABSI and atherogenic indices, so they can be considered mediator markers.

**Table 4:** Evaluation of the mediating effect of inflammatory markers in the relationship between ABSI and atherogenic indices.

**Atherogenic indices**	**inflammatory markers**	**ABSI higher** ≥**0.791**		
		β	**95% CI**	***P-*** **value**
AC	hs-CRP	0.11	−0.04 to 0.75	**0.08**
	PAI-1	0.17	0.06 to 1.06	0.02
	MCP-1	0.13	0.00 to 0.86	**0.05**
	TGF- β	0.59	−0.99 to 1.23	**0.06**
	Galectin-3	0.22	−0.03 to 1.40	**0.06**
CRI.I	hs-CRP	0.11	−0.04 to 0.75	**0.08**
	PAI-1	0.17	−0.009 to 0.007	0.02
	MCP-1	0.13	0.00 to 0.86	**0.05**
	TGF- β	0.06	−0.99 to 1.23	**0.81**
	Galectin-3	0.22	−0.03 to 1.4	**0.06**

ABSI, A Body Shape Index; AC, atherogenic coefficient; CRI, Cardiac Risk Index; TyG, triglyceride glucose; hs-CRP, high sensitivity C-reactive protein; PAI-1, plasminogen activator inhibitor-1; MCP-1, monocyte chemoattractant protein-1; TGF- β, transforming growth factor- β.

Adjusted model to total energy intake and age as covariates in addition to the inflammatory markers in the linear regression model.

The ABSI group <0.791 was considered the reference group. The bolded values are related to significant *p*-value >= 0.05.

hs-CRP was an intermediate for ABSI and AC (β = 0.11, 95%CI = −0.04 to 0.75, *P* = 0.08) and CRI-I (β = 0.17, 95%CI = 3.94 to 21.95, *P* = 0.53). MCP-1 was an intermediate agent for ABSI and AC and CRI-I (β = 0.13, 95%CI = 0.00 to 0.86, *P* = 0.05). TGF-β was an intermediate agent for ABSI and AC (β = 0.59, 95%CI = −0.99 to 1.23, *P* = 0.06) and CRI-I (β = 0.06, 95%CI = −0.99 to 1.23, *P* = 0.81). Galectin-3 was an intermediate agent for ABSI and AC (β = 0.22, 95%CI = −0.03 to 1.40, *P* = 0.06) and CRI-I (β = 0.22, 95%CI = −0.03 to 1.4, *P* = 0.06).

### 3.6. The association of BRI with metabolic markers and atherogenic indices

The association between BRI with metabolic markers and atherogenic indices is represented in the crude and adjusted models in Table 5.

**Table 5:** Association of BRI with metabolic markers and atherogenic indices.

**Metabolic markers**	**Models**	**BRI higher** ≥**5.642**		
		β	**95% CI**	***P*** **-value**
FBS (mmol/dl)	Crude	0.19	1.34 to 6.12	**0.002**
	Adjusted	0.18	1.81 to 5.99	**0.004**
TC (mg/dl)	Crude	0.19	4.86 to 22.45	**0.002**
	Adjusted	0.17	3.94 to 21.95	**0.005**
TG (mg/dl)	Crude	0.19	8.25 to 37.4	**0.002**
	Adjusted	0.19	8.19 to 38.52	**0.003**
HDL (mg/dl)	Crude	−0.03	−3.57 to 1.89	0.54
	Adjusted	−0.04	−3.78 to 1.87	0.50
LDL (mg/dl)	Crude	0.1	−1.08 to 1.89	0.10
	Adjusted	0.16	−1.71 to 10.35	0.09
**Atherogenic indices**				
AIP	Crude	0.18	0.15 to 0.9	**0.005**
	Adjusted	0.18	0.03 to 0.15	**0.004**
AC	Crude	0.17	0.15 to 0.9	**0.005**
	Adjusted	0.17	0.13 to 0.91	**0.008**
CRI-I	Crude	0.17	0.15 to 0.9	**0.005**
	Adjusted	0.17	0.13 to 0.91	**0.008**
CRI-II	Crude	0.13	0.01 to 0.3	**0.02**
	Adjusted	0.13	0.006 t0 0.3	**0.04**
TyG	Crude	0.14	0.03 to 0.3	**0.02**
	Adjusted	0.15	0.03 to 0.2	**0.02**

BRI, Body Roundness Index; FBS, fasting blood sugar; TC, total cholesterol; TG, triglyceride; HDL, high-density lipoprotein; LDL, low-density lipoprotein; AIP, atherogenic index of plasma; AC, atherogenic coefficient; CRI, Cardiac Risk Index; TyG, triglyceride glucose.

Metabolic markers and atherogenic indices are adjusted for age and total energy intake in the linear regression model.

BRI group 5.642 was considered the reference group. The bolded values are related to significant *p*-value = < 0.05.

In the crude model, there was a significant association between BRI with FBS (β = 0.19, 95%CI = 1.34 to 6.12, *P* = 0.002), TC (β = 0.19, 95%CI = 4.86 to 22.45, *P* = 0.002), TG (β = 0.19, 95%CI = 8.25 to 37.4, *P* = 0.002), AIP (β = 0.18, 95%CI = 0.15 to 0.9, *P* = 0.005), AC and CRI-I (β = 0.17, 95%CI = 0.15 to 0.9, *P* = 0.005), CRI-II (β = 0.13, 95%CI = 0.01 to 0.3, *P* = 0.02), and TyG (β = 0.14,95% CI = 0.03 to 0.3, *P* = 0.02).

After we adjusted for age and energy intake, there was a significant association between BRI with FBS (β = 0.18, 95%CI = 1.81 to 5.99, *P* = 0.004), TC (β = 0.17, 95%CI = 3.94 to 21.95, *P* = 0.005), TG (β = 0.19, 95%CI = 8.19 to 38.52, *P* = 0.003), and atherogenic risk factors including AIP (β = 0.18, 95%CI = 0.03 to 0.15, *P* = 0.004), AC and CRI.I (β = 0.17, 95%CI = 0.13 to 0.91, *P* = 0.008), CRI-II (β = 0.13, 95%CI = 0.006 t0 0.3, *P* = 0.04), and TyG (β = 0.13,95% CI = 0.03 to 0.2, *P* = 0.02) in participants with higher BRI scores compared to participants with lower BRI scores.

### 3.7. The evaluation of the mediating effect of inflammatory markers in the relationship between BRI with metabolic markers and atherogenic indices

We evaluated the effect of inflammatory markers including hs-CRP, PAI-1, MCP-1, TGF-β, and Galectin-3 as mediators for the association between BRI with metabolic markers and atherogenic indices (Table 6). Each of these inflammatory markers was included in the final model as a confounding variable, along with age and total energy intake. We observed that some of these inflammatory markers attenuated the relationship between BRI with metabolic markers and atherogenic indices, such that they can be considered as mediating markers.

**Table 6:** Evaluation of the mediating effect of inflammatory markers in the relationship between BRI with metabolic markers and atherogenic indices.

**Metabolic markers**	**Inflammatory markers**	**BRI higher** ≥**5.642**		
		β	**95% CI**	***P*** **-value**
FBS (mmol/dl)	hs-CRP	0.16	0.51 to 5.62	0.01
	PAI-1	0.1	−0.85 to 4.59	**0.17**
	MCP-1	0.15	0.44 to 5.53	0.02
	TGF- β	0.93	1.33 to 38.46	0.03
	Galectin-3	0.1	−2.73 to 7.53	**0.35**
TC (mg/dl)	hs-CRP	0.18	3.47 to 23.04	0.008
	PAI-1	0.19	3.09 to 24.93	0.01
	MCP-1	0.17	3.52 to 22.87	0.008
	TGF- β	0.59	−35.78 to 93.51	**0.33**
	Galectin-3	0.16	−4.23 to 27.01	**0.15**
TG (mg/dl)	hs-CRP	1.06	−5.99 to 144.83	**0.06**
	PAI-1	0.15	−0.02 to 36.46	**0.05**
	MCP-1	0.22	10.94 to 42.18	0.001
	TGF- β	0.15	1.75 to 34.92	0.03
	Galectin-3	0.29	9.81 to 70.76	0.01
**Atherogenic indices**				
AIP	hs-CRP	0.11	−0.01 to 0.12	**0.10**
	PAI-1	0.15	0.00 to 0.15	0.04
	MCP-1	0.21	0.04 to 0.17	0.002
	TGF- β	0.88	−0.08 to 0.82	**0.09**
	Galectin-3	0.31	0.04 to 0.31	0.008
AC	hs-CRP	0.04	−0.005 to 0.08	**0.08**
	PAI-1	0.5	0.001 to 1.01	**0.05**
	MCP-1	< 0.001	−0.003 to 0.002	**0.68**
	TGF- β	0.003	−0.001 to 0.007	**0.15**
	Galectin-3	0.76	0.03 to 1.49	0.04
CRI-I	hs-CRP	0.11	−0.04 to 0.81	**0.09**
	PAI-1	0.15	0.001 to 1.01	**0.05**
	MCP-1	0.18	0.17 to 1.03	0.006
	TGF- β	0.68	−0.5 to 2.63	**0.15**
	Galectin-3	0.24	0.03 to 1.49	0.04
CRI-II	hs-CRP	0.02	−0.12 to 0.19	**0.67**
	PAI-1	0.06	−0.09 to 0.25	**0.38**
	MCP-1	0.14	0.01 to 0.32	0.03
	TGF- β	0.81	−0.47 to 1.89	**0.20**
	Galectin-3	0.22	−0.01 to 0.67	**0.05**
TyG	hs-CRP	0.11	−0.02 to 0.35	**0.09**
	PAI-1	0.13	−0.02 to 0.39	**0.08**
	MCP-1	0.19	0.07 to 0.44	0.006
	TGF- β	0.85	−0.23 to 1.22	**0.15**
	Galectin-3	0.27	0.06 to 0.75	0.02

BRI, Body Roundness Index; FBS, fasting blood sugar; TC, total cholesterol; TG, triglyceride; AIP, atherogenic index of plasma; AC, atherogenic coefficient; CRI, Cardiac Risk Index; TyG, triglyceride glucose; hs-CRP, high sensitivity C-reactive protein; PAI-1, plasminogen activator inhibitor-1; MCP-1, monocyte Chemoattractant Protein-1; TGF-, transforming growth factor- β.

Adjusted model to total energy intake and age as covariates in addition to the inflammatory markers in the linear regression model.

BRI group <5.642 was considered the reference group. The bolded values are related to significant *p*-value >= 0.05.

Regarding BRI, hs-CRP was an intermediate marker of this index and TG (β = 1.06, 95%CI = −5.99 to 144.83, *P* = 0.06), AIP (β = 0.11, 95%CI = −0.01 to 0.12, *P* = 0.10), AC (β = 0.17, 95%CI = 3.94 to 21.95, *P* = 0.05), CRI-I (β = 0.11, 95%CI = −0.04 to 0.81, *P* = 0.09), CRI-II (β = 0.02, 95%CI = −0.12 to 0.19, *P* = 0.67), and TyG (β = 0.11, 95%CI = −0.02 to 0.35, *P* = 0.09).

PAI-1 was an intermediate for BRI and FBS (β = 0.1, 95%CI = −0.85 to 4.59, *P* = 0.17), TG (β = 0.15, 95%CI = 3.94 to 21.95, *P* = 0.05), AC (β = 0.05, 95%CI = 0.001 to 1.01, *P* = 0.05), CRI-I (β = 0.15, 95%CI = 0.001 to 1.01, *P* = 0.05), CRI-II (β = 0.06, 95%CI = −0.09 to 0.25, *P* = 0.38), and TyG (β = 0.13, 95%CI = −0.02 to 0.39, *P* = 0.08).

MCP-1 was an intermediate for BRI and AC (β < 0.001, 95%CI = 3.94 to 21.95, *P* = 0.68). TGF-β was an intermediate agent for BRI and TC (β = 0.59, 95%CI= −35.78 to 93.51, *P* = 0.33), AIP (β=0.88, 95%CI = −0.08 to 0.82, *P* = 0.09), AC (β = 0.003, 95%CI = −0.001 to 0.007, *P* = 0.15), CRI-I (β = 0.68, 95%CI = −0.5 to 2.63, *P* = 0.15), CRI-II (β = 0.81, 95%CI = −0.47 to 1.89, *P* = 0.¬20), and TyG (β = 0.85, 95%CI = −0.23 to 1.22, *P* = 0.15).

Galectin-3 was an intermediate for BRI and FBS (β = 0.1, 95%CI = −2.73 to 7.53, *P* = 0.35), TC (β = 0.16, 95%CI = −4.23 to 27.01, *P* = 0.15), and CRI-II (β = 0.22, 95%CI = −0.01 to 0.67, *P* = 0.05).

## 4. Discussion

In the current cross-sectional study, we investigated inflammatory markers' mediatory effect on the association between ABSI and BRI with cardiometabolic risk factors in overweight and obese women. To the best of our knowledge, this study is the first to investigate such a mediatory effect. The present study revealed that, after adjusting for age and total energy intake, there was a significant positive association between ABSI and AC and CRI-I. We also revealed that this association could be influenced by the mediation of inflammatory markers, such as hs-CRP, MCP-1, TGF-β, and Galectin-3. Moreover, we observed a significant positive association between BRI and FBS, TC, TG, and atherogenic indices, after adjusting for confounding variables. In this regard, the association between BRI and AC, CRI-I, CRI-II, and TyG appears to be influenced by hs-CRP, PAI-1, hs-CRP, and TGF-β.

The results of previous studies have shown a significant relationship between ABSI and BRI with cardiometabolic risk factors, which are more accurate indicators than traditional measures, e.g., BMI (11–13). For ABSI, we observed a positive relationship with AC and CRI-I, two atherogenic indices. Concordant with our results, some studies concluded that high ABSI is associated with an increased risk of CVD (32, 33). The positive association of ABSI with small dense LDL, TG, and FBS and the negative association with HDL have been shown in previous studies (27, 34). Indeed, one study highlighted that ABSI can be a valuable index for evaluating central obesity in cardiometabolic risk (27); however, another study did not show this (14). The correlation between ABSI and visceral adipose tissue (VAT) was demonstrated in another study (35), and it was also reported that higher ABSI is associated with higher adipose tissue accumulation around the abdomen (36). A retrospective study concluded that using ABSI and BMI together is more associated with VAT thickness than BMI alone (27). Considering the relationship between ABSI and cardiometabolic risk factors, inflammation may play an effective role. As our study demonstrated, inflammatory markers, including hs-CRP, MCP-1, TGF-β, and Galectin-3, have a mediatory effect on the association of ABSI with AC and CRI-I. Indeed, central obesity can decrease plasma adiponectin via an increase in the pro-inflammatory adipokines (37). Leptin and adiponectin, as energy-regulating hormones, are released from adipose tissues, and their secretion increases and decreases, respectively, in MetS/obese patients. Leptin is negatively associated with HDL and adiponectin (38), while, in obese patients, increased levels of hs-CRP have been observed. High hs-CRP is associated with low adiponectin, and thus, reduced adiponectin production has been suggested to cause vascular and systemic inflammation in obese patients (39).

In addition to CVD, some evidence has suggested that BRI could be a good predictor for metabolic syndrome and insulin resistance (11, 14, 15, 26). One study stated that BRI can predict both CVD and CVD risk factors (11). We observed a significant association between BRI and cardiometabolic risk factors in the present study. BRI is established as an index of body fat distribution, has been reported to be a good predictor of body fat and VAT percent, and is more accurate than BMI, WC, and WHR because of the higher health status reflection (40). Recently, a study showed that BRI, as an index of adipose tissue, can indicate the presence of left ventricular hypertrophy (41) and may be a more helpful predictor of MetS and IR than BMI (15). The results of the aforementioned study demonstrated a significant association between BRI with TG, HDL, LDL, BP, FBS, and inflammatory markers (15). Both BRI and ABSI, as well as WC, can predict inflammation levels, based on hs-CRP levels, in obese patients (15). Similar to ABSI, we observed that inflammatory markers can also mediate the relationship between BRI and cardiometabolic risk factors. We observed that inflammation mediates the association between BRI and FBS, TC, TG, and atherogenic indices. Among the mechanisms that can be mentioned are the following: first, the breakdown of VAT through many steps can increase gluconeogenesis in the liver, hence reducing the very low-density lipoprotein (VLDL) output; this leads to impaired glucose tolerance and increased TG (42–45). Second, in inflammatory conditions, adipose tissue begins to release fat and produce cortisol and pro-inflammatory cytokines, such as adipokines, which itself affects metabolic health (42, 43).

The present study has some strengths that should be noted. First, to the best of our knowledge, this is the first study to evaluate the mediatory effect of inflammatory markers on the association between ABSI and BRI with cardiometabolic risk factors in overweight and obese women. Second, this study was conducted on obese and overweight Iranian women, yielding a detailed insight into this population. However, this study also has limitations that must be acknowledged. First, the relatively small sample size in this study clearly represents an avenue for improvement in future studies. Second, the cross-sectional design of the study precludes causal inferences being made. Third, to obtain the usual dietary intake, we used the FFQ, which is based on participants' memory, and may be subjected to recall bias. Fourth, some errors outside the operational control of the study while measuring may have occurred.

## 5. Conclusion

In summary, the present study found a significant positive association between ABSI and atherogenic indices. Moreover, we observed a significant positive association between BRI and FBS, TC, TG, and atherogenic indices. We revealed that these associations could be influenced by the mediation of inflammatory markers, including hs-CRP, MCP-1, TGF-β, and Galectin-3. However, the cross-sectional nature of our study does not allow us to attribute causality among these relationships. Further prospective studies with larger sample sizes are needed to better clarify this issue.

## Data availability statement

The data analyzed in this study is subject to the following licenses/restrictions: The data that support the findings of this study are available from KM but restrictions apply to the availability of these data, which were used under license for the current study, and so are not publicly available. Data are however available from the authors upon reasonable request and with permission of KM. Requests to access these datasets should be directed to mirzaei_kh@sina.tums.ac.ir.

## Ethics statement

The studies involving human participants were reviewed and approved by the study protocol was approved by the Ethics Committee of Tehran University of medical sciences (IR.TUMS.VCR.REC.1397.804) and is acknowledged by authors. All participants signed a written informed consent. The patients/participants provided their written informed consent to participate in this study.

## Author contributions

AM contributed to the conception and design. NV, MG, DK, CC, MR, and AM contributed to all experimental work. FA contributed to data and statistical analysis. KM supervised the whole project. All authors performed editing, approved the final version of this study for submission, participated in the finalization of the manuscript, approved the final draft and read and approved the final manuscript.
